# Hypnic Jerks, Major Depressive Disorder, and Antidepressant Use: A Possible Relationship

**DOI:** 10.7759/cureus.47436

**Published:** 2023-10-21

**Authors:** Saleh A. Alghamdi

**Affiliations:** 1 Clinical Neurosciences Department, Imam Mohammad Ibn Saud Islamic University, Riyadh, SAU

**Keywords:** antidepressant, clonazepam, depressive disorder, sleep problems, onset insomnia, hypnic jerks

## Abstract

The term hypnic jerks (also known as sleep starts or hypnagogic jerks) refers to a physiological phenomenon that accompanies sleep-wake transitions and can occur in healthy individuals of any age.

Various physiological and stressful stimuli can precipitate hypnic jerks and contribute to their frequency and amplitude, e.g., stress, fatigue, stimulants like caffeine, and certain medications.

Hypnic jerks are usually benign, but they can be intensified in certain situations, interfering with sleep onset and causing insomnia. Here we reported the case of a patient who suffered from intensified hypnic jerks that led to the development of major depressive disorder. Although the depressive symptoms improved on escitalopram 20 mg, the hypnic jerks increased significantly. Clonazepam was found to be very effective in reducing the hypnic jerks and stabilizing his condition.

## Introduction

Hypnic jerks, sleep starts, or hypnic myoclonia are a physiological, non-periodic, and abrupt myoclonic muscle contraction involving nearly all body muscles that occurs mainly on falling asleep. These physiological phenomena are experienced by up to 70% of the adult population sometime in their lives [1].

They presumably arise from sudden descending volleys that originate in the brainstem reticular formation and are activated by the instability of the system during the transition between wake and sleep [2].

They are associated with autonomic activation, resulting in tachycardia, tachypnea, and sudomotor activity described as a shock or falling feeling [3].

Hypnic jerks may be exacerbated during stressful conditions occurring during a normal part of sleep onset [4]. These stressful conditions that may trigger hypnic jerks include fatigue, stress, sleep deprivation, vigorous exercise, and stimulants like caffeine and nicotine [5].

Hypnic jerks, as per the second International Classification of Sleep Disorders (ICSD-2), are classified as isolated symptoms, apparently normal variables, and unresolved issues. They fall on the borderline between normal and abnormal sleep.

However, these are benign phenomena, but they need to be treated if they interfere with one's sleep or lead to a significant reduction in the quality of life [6].

## Case presentation

A middle-aged male employee visited our outpatient psychiatry department at Saudi German Hospital in Riyadh, Saudi Arabia. For nearly four years, he has complained of ongoing sadness and decreased interest in daily activities. Additionally, he became withdrawn and lost interest in the social activities he formerly enjoyed. His appetite diminished, and he lost weight. However, there was no history of suicidal ideation, suicide attempts, or self-harming behavior. The patient attributed his symptoms to an issue with sleep that he has had for the previous two years. He reported experiencing recurrent, sudden, whole-body muscle contractions at the onset of falling asleep that would wake him up, followed by difficulty falling back asleep. It was accompanied by heart palpitations, a tense feeling, and an excessive fear of not being able to sleep again. The next day, his fatigue would negatively impact his work performance. No history of snoring, cessation of breathing, excessive dreaming, sleepwalking or talking. No history of hallucinations, or ictal sensations prior to or following sleep.

Except for escitalopram 20 mg, which he took for four years, he visited a number of psychiatric clinics and used a number of psychotropic drugs (e.g., paroxetine, mirtazapine, quetiapine, and olanzapine only for less than two months for each). The escitalopram had slightly improved his mood symptoms but had exacerbated his sleep condition. There were, however, no manic, psychotic, obsessive-compulsive, or substance use disorder symptoms observed.

His previous medical and surgical history was of no importance. His family had no history of psychiatric disorders.

On examination of his mental status, he appeared to be his stated age, was slim (body mass index (BMI) = 17.7), cooperative, and properly groomed, but appeared exhausted. His mood was sad and restricted. He expressed feelings of hopelessness and helplessness but denied having suicidal thoughts. There were no psychotic symptoms detected.

According to the criteria of the Diagnostic and Statistical Manual of Mental Disorders, Fifth Edition, he was diagnosed with major depressive disorder and hypnic jerks, and a sleep disorder was to be ruled out. Due to his previous negative experience with medication changes, he was hesitant to switch antidepressants. However, he asked if we could help him with his sleep problem rather than his mood symptoms.

He was given 10 mg of zolpidem at bedtime as needed for one month. Despite the fact that he regularly used it before going to sleep, it only offered him partial relief, enabling him to sleep without interruption for about two to three hours each night. However, he started experiencing headaches upon waking, which prompted him to discontinue its use.

He was referred to a sleep medicine clinic in order to rule out the possibility of a sleep disorder such as restless legs syndrome, periodic limb movement sleep disorder, or any other sleep-related movement disorder. A polysomnogram was performed, and no anomalous sleep events were detected. His physician has advised him to continue taking the same medications. A neurologist screened the patient for any potential neurological diseases. There was no history of altered level of consciousness, foaming at the mouth, upward rolling of the eyeballs, loss of sphincter control, or aberrant involuntary movements during the day, and other specific neurological diagnoses were ruled out. The diagnosis of intensified hypnic jerk was confirmed, and clonazepam 0.5 mg PO at bedtime was administered.

On regular follow-up, in one month he showed dramatic improvement in his condition, and the hypnic jerk movement disappeared completely at night. Interestingly, he continued to experience the hypnic jerks when he tried to get day naps.

The depressive symptoms also vanished, and we discontinued escitalopram 20 mg in favor of clonazepam 0.5 mg PO at bedtime as needed and another 0.5 mg PO for day naps if required. He attempted to reduce the nightly dosage of clonazepam from 0.5 mg to 0.25 mg; however, it was found to be less efficacious.

The patient concluded two years on this regimen without experiencing a recurrence of mood or sleep symptoms, nor did he develop tolerance to clonazepam. As agreed with the patient, the future plan is to progressively wean him off clonazepam in order to give him a trial without medication.

Consent has been obtained from the patient to report his case.

## Discussion

Hypnic jerks are a common physiological phenomenon, and their course is usually benign and resolves without any neurological sequelae. They are actually classified within Section VII of the ICSD-2, which includes ‘‘isolated symptoms, apparently normal variants, and unresolved issues,’’ frequently occurring in normal people and at any age [5]. Recent studies suggest, however, that hypnic jerks may be a symptom of certain diseases, are more common in chronic health conditions that disrupt sleep, and may be a symptom of other movement disorders. It is important to figure out the differential diagnosis, including nocturnal seizures, nonepileptic seizures, additional parasomnias, hyperekplexia, restless legs syndrome (RLS), periodic limb movement disorder (PLMD), excessively fragmented myoclonus, and mental diagnoses [7].

The intensified hypnic jerks are mainly determined by the patient's medical history and physical examination. Polysomnography is useful for excluding other sleep disorders. Though an adequate explanation and reassurance may be sufficient, some patients may require a small dose of clonazepam (0.5-1 mg at bedtime) to ameliorate the symptoms on a short-term basis [5].

The intensified hypnic jerks can cause chronic sleep deprivation, potentially resulting in depression as a consequence of neurochemical changes in the brain [8]. Therefore, early diagnosis and treatment are of utmost importance.

## Conclusions

Hypnic jerk is a prevalent sleep-related movement that is, in the majority of cases, benign. However, it can cause significant sleep disturbances, daytime fatigue, a substantial reduction in quality of life, and an increase in the risk of major depressive disorder in the patient. It can be managed through reassurance, education, and lifestyle modifications. A small dosage of clonazepam (0.5-1 mg at bedtime) was observed to be highly beneficial in severe cases, as evidenced by this case report and numerous others. The development of tolerance, however, should be noted.

Furthermore, due to the reliance on a single case report, the generalizability of the finding was limited. To ascertain the applicability and generalizability of these findings, much larger sample sizes and statistical analysis would be necessary.
